# Bioavailable Concentrations of Delphinidin and Its Metabolite, Gallic Acid, Induce Antioxidant Protection Associated with Increased Intracellular Glutathione in Cultured Endothelial Cells

**DOI:** 10.1155/2017/9260701

**Published:** 2017-09-07

**Authors:** Katarzyna Goszcz, Sherine J. Deakin, Garry G. Duthie, Derek Stewart, Ian L. Megson

**Affiliations:** ^1^Department of Diabetes & Cardiovascular Science, University of the Highlands & Islands, Centre for Health Science, Inverness IV2 3JH, UK; ^2^Rowett Institute of Nutrition & Health, University of Aberdeen, Foresterhill, Aberdeen AB25 2ZD, UK; ^3^James Hutton Institute, Invergowrie, Dundee DD2 5DA, UK; ^4^School of Engineering and Physical Sciences, Heriot Watt University, Edinburgh EH14 4AS, UK

## Abstract

Despite limited bioavailability and rapid degradation, dietary anthocyanins are antioxidants with cardiovascular benefits. This study tested the hypothesis that the antioxidant protection conferred by the anthocyanin, delphinidin, is mediated by modulation of endogenous antioxidant defences, driven by its degradation product, gallic acid. Delphinidin was found to degrade rapidly (t1/2 ~ 30 min), generating gallic acid as a major degradation product. Both delphinidin and gallic acid generated oxygen-centred radicals at high (100 *μ*M) concentrations *in vitro*. In a cultured human umbilical vein endothelial cell model of oxidative stress, the antioxidant protective effects of both delphinidin and gallic acid displayed a hormesic profile; 100 *μ*M concentrations of both were cytotoxic, but relatively low concentrations (100 nM–1 *μ*M) protected the cells and were associated with increased intracellular glutathione. We conclude that delphinidin is intrinsically unstable and unlikely to confer any direct antioxidant activity *in vivo* yet it offered antioxidant protection to cells at low concentrations. This paradox might be explained by the ability of the degradation product, gallic acid, to confer benefit. The findings are important in understanding the mode of protection conferred by anthocyanins and reinforce the necessity to conduct *in vitro* experiments at biologically relevant concentrations.

## 1. Introduction

Anthocyanins are polyphenolic phytochemicals that, although not essential for survival, are among the factors that contribute to the health benefits of a fruit-rich diet. From a cardiovascular perspective, polyphenols have been associated with a reduction in mortality associated with heart disease, a lower incidence of myocardial infarction [1, 2], reduced blood pressure [3–5], and protection against atherosclerosis [6–10]. The mechanisms through which phenolics exert their cardioprotective actions are not yet fully understood, but protection against oxidative damage and modulation of vascular function is strongly implicated [5, 9, 11–17].

Reactive oxygen species (ROS) play an important role in cardiovascular disease [11, 18, 19]. Oxygen-centred free radicals have a cytotoxic and proinflammatory effect on endothelial cells that line blood vessels [20, 21]; superoxide (O_2_^∙−^) readily reacts with the cardioprotective agent, NO, generating highly cytotoxic peroxynitrite (ONOO^−^; [22]). Furthermore, ROS-mediated oxidation of critical lipids, and low-density lipoprotein (LDL) in particular, is a key event in the pathogenesis of atherosclerosis [23]. ROS are also prothrombotic via promotion of platelet activation [18]. Taken together, these characteristics of ROS impact on all of the critical stages of atherothrombotic disease, from endothelial dysfunction and inhibition of protective NO to oxidative modification of LDL and promotion of inflammation and thrombosis [19]. Elimination of ROS by antioxidants is an attractive therapeutic option.

Anthocyanins are abundant in a wide range of berries (e.g., blackberries, blackcurrants, raspberries, red grapes, cranberries, and elderberries) and are considered to be strong antioxidants on account of their polyphenolic structure [7, 24–30]. At least some of the cardiovascular benefits of red wine are mediated by anthocyanins, particularly delphinidin [12, 17, 31, 32]. A link has long been forged between the antioxidant potential of anthocyanins and their protective effects in cardiovascular health, but they are characterised by low bioavailability (~1 *μ*M) and the concentrations required to induce direct antioxidant activity are unachievable *in vivo* [12, 14, 33–38]. This aspect is often cited as a major flaw in the argument that polyphenols modulate the benefits attributed to red wine.

Berry-derived anthocyanins are typically found in the more stable glycosylated form, but after consumption, they are subjected to digestive processes that deprive them of the sugar moiety, releasing the less stable aglycone [34, 36]. Under physiological conditions, sugar-free anthocyanins can degrade further into smaller phenolic compounds (e.g., phenolic acids and aldehydes [39–42]). The form in which they are absorbed and exist in the bloodstream remains unclear, but it is likely that the degradation products (simple phenolic acids and/or aldehydes) might predominate over the parent compounds [11, 40]. This detail is important because phenolic compounds have been shown to have paradoxical prooxidant properties [33, 43–46], a concept that would throw into question how ingestion of the parent polyphenols could confer direct antioxidant activity *in vivo*.

It is becoming increasingly recognised that phenolic compounds, including anthocyanins, can interact with various molecular targets and affect multiple signalling pathways, providing an alternative putative mechanism to confer antioxidant activity which is consistent with the low concentrations found *in vivo* [13, 47–55]. This study set out to test the hypothesis that the protective effects of biologically relevant concentrations of the anthocyanin, delphinidin, in cultured endothelial cells are mediated by upregulation of endogenous antioxidant defences by the primary metabolite, gallic acid.

## 2. Materials and Methods

### 2.1. Materials

Chemicals, reagents, and consumables were purchased from the following manufacturers: delphinidin chloride (Extrasynthese, Genay, France); DMSO, ascorbic acid, formic acid, gallic acid, gelatin solution (type B, 2% in H_2_O), endothelial cell growth supplement, sodium pyruvate, HEPES solution, 0.5% trypsin/EDTA (1X), iron(lll) chloride (FeCl_3_), 2,4,6-tri(2-pyridyl)-*s*-triazine (TPTZ), and ferrous sulfate heptahydrate (FeSO_4_·7H_2_O) (Sigma-Aldrich, Poole, Dorset, UK); basal medium M199 (+Earle's, +L-glutamine, and +phenol red) and basal medium M199 (+Earle's, +L-glutamine, −phenol red, and +bicarbonate) (Invitrogen, Paisley, UK); pyrogallol, methanol, acetonitrile, ethanol, acetone, Coomassie (Bradford) reagent kit, sodium acetate, and hydrochloric acid (Thermo Fisher Scientific Ltd., Loughborough, UK); Tempone-H hydrochloride and ROS-ID® Total ROS/Superoxide detection kit (ENZO Life Sciences Ltd., Exeter, UK); PBS (without Ca and Mg), foetal bovine serum, penicilin/streptomycin (100X), and bovine serum albumin (PAA Laboratories GmbH, Pasching, Austria); HUVECs (Cell Applications Inc., San Diego, CA, USA); heparin sodium (Wockhardt Ltd., Wrexham, UK); GSH, catalase, and SOD assay (Cayman Chemical Company, Ann Arbor, MI, USA); and filter vials (Thomson single StEP standard filter vials) (Thomson Instrument Company, Oceanside, CA, USA).

### 2.2. Determination of Delphinidin Degradation Profile and Products in Tissue Culture Medium

#### 2.2.1. Spectrophotometry

It was important to understand the degradation pattern of delphinidin under our experimental conditions at the outset in order to inform the subsequent functional experiments. Delphinidin (200 *μ*M; the lowest concentration easily measurable by spectrophotometry) decomposition was tested in phenol red-free tissue culture medium (pH 7.4, 37°C) containing 0.2% DMSO, alone or in the presence of the superoxide generator, pyrogallol (100 *μ*M), or the antioxidant and reducing agent, ascorbic acid (5 mM). Delphinidin is subject to a bathochromic shift—a change in absorption peak to a higher value, dependent on the environmental conditions [56]. Absorbance (*λ* = 585 nm) was therefore measured (every 10 minutes for the first hour, then every hour for 7 consecutive hours, and finally at 24 h) using a plate reader (Varioskan Flash with operating software SkanIt version 2.4; Thermo Fisher Scientific Ltd., Loughborough, UK; pre-heated to 37°C). Each experiment was performed on three separate occasions (*n* = 3).

#### 2.2.2. LC-MS/MS

Delphinidin (100 *μ*M) was incubated in tissue culture medium for 30 minutes (humidified incubator (Heracell 150 CO_2_, Thermo Fisher Scientific, Loughborough, UK), 37°C, pH 7.4, 5% CO_2_, in the dark). Control samples were treated with vehicle only (0.2% (*v/v*) DMSO). All samples were collected after 30 min and extracted as follows: proteins were precipitated from the collected tissue culture medium (1 ml) by addition of an equal volume of extraction buffer (ice cold 100% methanol containing 0.2% formic acid (*v/v*)). Samples were kept on ice for 1 h, prior to centrifugation (5 min, room temperature, 10,000*g*). The supernatant was removed and kept on ice. The pellet was resuspended in ice cold 50% methanol containing 0.1% formic acid (*v/v*) and centrifuged as before. The extraction procedure was repeated three times in order to extract as many phenolic compounds as possible. All supernatants were combined and centrifuged once more (3 min, 5000*g*). The collected supernatants were kept at −80°C prior to analysis using liquid chromatography-mass spectrometry (LC-MS/MS).

The volume of extracts was reduced to dryness by evaporation in a vacuum concentrator (SpeedVac, Thermo Fisher Scientific Ltd., Loughborough, UK; no heat) prior to resuspension in 100 *μ*l of 50% methanol containing 0.1% formic acid (*v/v*). Samples were transferred into filter vials (polytetrafluoroethylene, 0.45 *μ*m membranes) prior to analysis by LC-MS/MS (LTQ Orbitrap XL LC-MS fitted with an Acella 600 Pump, Acella photodiode array detector (PDA) detector, and Acella autosampler) in both positive and negative ion modes. The PDA scanned the wavelength range *λ* = 200–600 nm. There were two scan events: Fourier transform mass spectrometer full scan (80–2000 m/z) analysis was followed by data-dependent MS/MS of the most intense ions using a normalised collision energy of 45%. The capillary temperature was set at 300°C, with sheath gas at 40 psi and auxiliary gas at 5 psi. Samples (8 *μ*l) were eluted over a gradient of 0.1% formic acid in ultrapure water (A), 0.1% formic acid in 50% aqueous acetonitrile (B), and 0.1% formic acid in 95% aqueous acetonitrile (C) on a C18 column (50 × 2.1 mm, 1.9 Hypersil GOLD) at a flow rate of 700 *μ*l/min. Exact mass data were analysed using the resident Xcalibur Qual Browser software. The experiment was performed on 3 separate occasions (*n* = 3).

### 2.3. Antioxidant Activity: FRAP Assay

Antioxidant potential was measured using the ferric reducing ability of plasma (FRAP) assay according to the procedure described by Benzie and Strain [57], with minor modifications. Briefly, 100 *μ*l of 1% (*v/v*) test sample was mixed with 900 *μ*l of freshly prepared FRAP reagent, consisting of 20 mM ferric chloride (in water), 10 mM TPTZ (in 40 mM HCl), and 300 mM sodium acetate buffer (pH 3.6) in a volume ratio of 1 : 1 : 10, respectively. Absorbance after a 4 min incubation was measured at *λ* = 593 nm by spectrophotometry (Ultrospec 2100 pro; GE Healthcare Life Sciences, Little Chalfont, UK; [57]). Ferrous sulphate (FeSO_4_, in water) was used as a reference standard.

### 2.4. Prooxidant Measurements: EPR Spectrometry

**Figure 1:**
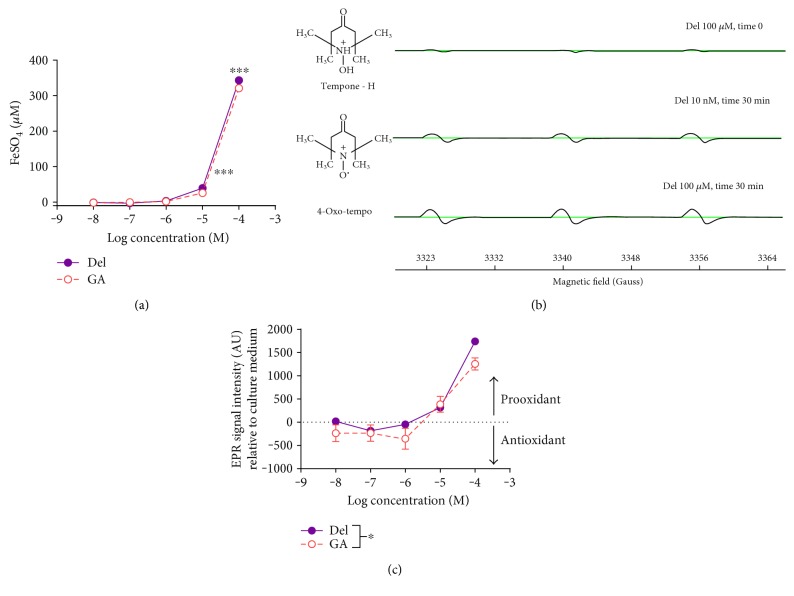
(a) Ferric reducing ability of plasma (FRAP) of delphinidin (Del) and gallic acid (GA) in culture medium (*n* = 3 for each phenolic; ^∗∗∗^*P* < 0.001, 2-factor ANOVA with Bonferroni posttest). (b) Electron paramagnetic resonance spectra of delphinidin samples (1 nM and 100 *μ*M) in tissue culture medium containing 2 mM Tempone-H at time 0 and 30 min. Addition of a superoxide radical (produced by delphinidin) to the EPR-silent spin trap (Tempone-H) forms a persistent adduct (4-oxo-tempo) which is EPR active and displays multiline spectrum characteristic of an unpaired electron in the vicinity of a nucleus. (c) Rate of oxygen-centred free radical production by Del and GA in tissue culture medium. Prooxidant activity: tested compound generated more radicals than tissue culture medium alone. Antioxidant activity: tested compound reduced the amount of free radicals produced by medium alone (*n* = 5 for each phenolic; ^∗^*P* < 0.05, 2-factor ANOVA).

Electron paramagnetic resonance (EPR) is a spectrometric technique that facilitates detection of chemical species that possesses one or more unpaired electrons, such as free radicals and transition metal ions. EPR spectrometry detects free radicals unambiguously [58]. It measures the absorption of energy by unpaired electron(s) of the free radical species in the presence of magnetic field. Free radicals, however, are highly reactive chemical species and are very short-lived as a result. Therefore, we used the EPR spin-trapping technique in order to determine the radical production by selected phenolic compounds. EPR spin trapping is a method that is based on the reaction of a free radical with an EPR-silent spin trap. The product of this reaction, the EPR-active adduct, is more stable than the parent radical and exhibits characteristic multilined EPR spectrum (Figure 1(b)) [59]. The EPR spectrum of a single unpaired electron consists only of one line. Multilined EPR spectra are generated as a consequence of the interaction between the magnetic spin of the unpaired electron and the nuclear spin of a neighbouring nucleus within the spin trap; this is called hyperfine splitting (Figure 1(b)) [59, 60]. Tempone-H (1-hydroxy-2,2,6,6-tetramethyl-4-oxo-piperidine hydrochloride) is frequently used in detection and quantifying of oxygen-centred radicals such as superoxide and hydroxyl radicals [61–63] and was used here in order to assess the prooxidant potential of delphinidin and gallic acid. The technique cannot discriminate between different oxygen-centred radicals but excludes nonradical species and, through choice of spin-trap, can select for a specific class of radical species (e.g., Tempone-H for oxygen-centred radicals).

Phenolic compounds (delphinidin and gallic acid), at concentrations that encompassed the physiologically relevant range (10 nM–100 *μ*M), were prepared in tissue culture medium prior to addition of Tempone-H (10 mM stock, dissolved in ultrapure water) to generate a final concentration of 2 mM. 50 *μ*l volumes of samples were drawn into a glass capillary tubes and inserted into an EPR spectrometer (Miniscope MS200 spectrometer, Magnettech, Germany, with the following parameter settings: magnetic field (B_0_) 3343.48 G, sweep 49.10 G, sweep time 60 sec, smooth 1 sec, 4096 steps, modulation 2000 mG, microwave attenuation 10 dB, and gain 2 × 10^1^). EPR spectra were acquired as first derivatives.

Formation of 4-oxo-tempo adduct generates a characteristic 3-line EPR spectrum centred at 3340 Gauss. Peak intensity is proportional to the concentration of adduct formed (Figure 1(b)). Pro-/antioxidant activity of tested compounds was determined by measuring 4-oxo-tempo signal intensity generated after a 30 min incubation period (37°C in the dark) in tissue culture medium containing delphinidin or gallic acid. Tissue culture medium alone acted as a control. Prooxidant activity of phenolic compounds corresponded to an increase in spin adduct formation compared to vehicle, whereas antioxidant activity resulted in reduced radical formation compared to vehicle.

### 2.5. Human Umbilical Vein Endothelial Cell (HUVEC) Culture

HUVECs were propagated from pooled primary cultures of human umbilical cords, prescreened for VEGF-R2, Etk/Bmx, eNOS, Tie2, and Axl (Cell Applications Inc., San Diego, CA, USA), and supplied by the European Collection of Cell Cultures. HUVECs were subcultured according to the supplier's protocol, with minor modifications. Briefly, the cells were grown in flasks or on plates coated with gelatin (diluted 1 : 1 (*v/v*) with PBS), in growth medium, prepared according to the recipe described previously [64]. Basal medium M199 (+Earle's and +L-glutamine) was supplemented with 20% (*v/v*) foetal bovine serum, 100 *μ*g/ml penicillin/streptomycin, 20 *μ*g/ml endothelial cell growth supplement, 10 *μ*g/ml heparin, 20 mM HEPES, and 2 mM sodium pyruvate as an additional source of energy. HUVECs were grown in a humidified incubator (Heracell 150 CO_2_, Thermo Fisher Scientific, Loughborough, UK) at 37°C, 5% CO_2_, and passaged every 3-4 days, until they reached 80% confluence, determined by visual assessment. During subculturing, the cells were lifted using Trypsin/EDTA (0.05%/0.02%) and gentle agitation. Cells were counted using a haemocytometer and plated in 75 cm^2^ flasks (7.5 × 10^5^ cells) for further culture, or onto 6-well and 24-well plates (1 × 10^5^ and 2 × 10^4^ cells per well, resp.) for experiments. All studies were performed on 80% confluent HUVEC cultures at the sixth passage.

### 2.6. Cell Treatment—Phenolic and Oxidant Preparation

Samples containing phenolic compounds (1 nM–100 *μ*M) were prepared in warmed (37°C) phenol red-free tissue culture medium. Additionally, some of the delphinidin samples were incubated in culture medium for 1 h prior to application to cells in order to initiate delphinidin degradation and to obtain a mixture consisting of native delphinidin and its degradation products. The obtained mixture is referred to as “aged delphinidin” for use in subsequent cell culture experiments.

Three cell culture models of oxidative stress were used in the study: pyrogallol (superoxide radical generator, O_2_^∙−^; [65]), hydrogen peroxide (H_2_O_2_; [66]), and pyocyanin (intracellular O_2_^∙−^ generator, via induction of mitochondrial dysfunction; [67]). All oxidants were prepared in warmed (37°C) phenol red-free tissue culture medium deprived of sodium pyruvate, which is known hydrogen peroxide scavenger, therefore not appropriate for oxidative stress model studies [68].

Due to the fact that HUVECs were pooled from different donors, their susceptibility to oxidants varied from batch to batch. The concentrations of oxidising agents were therefore optimised prior to test experiments for each batch of cells, in order to select an appropriate concentration of each oxidant that caused a submaximal, but easily detectable loss of cell viability after 24 h (20–70%). Concentrations of pyrogallol used in test experiments varied from 100 *μ*M to 180 *μ*M.

### 2.7. Assessment of Cell Viability: Trypan Blue Exclusion and MTT Assay

The Trypan blue exclusion assay was used in some studies for routine cell counting but was also used to determine the level of cytotoxicity of the test phenolic agents. Following trypsinization, Trypan blue (0.1% *v/v*) was added to the cell culture well (1 : 1, 5 min) prior to cell counting (stained and unstained) using a haemocytometer. The amount of live (Trypan blue negative) and dead (Trypan blue positive) cells was expressed as the number of stained or unstained per ml.

The 3-(4,5-dimethylthiazol-2-yl)-2,5-diphenyltetrazolium bromide (MTT) reduction assay is a metabolic activity assay that is frequently used to measure cell viability. Viable cells with active metabolism reduce yellow MTT to stable, purple, coloured formazan crystals, which can be dissolved. Dead cells, on the other hand, lose the ability to convert MTT into formazan product. The purple solution is measured spectrophotometrically, and its formation is a suitable indicator of cell viability. The mechanism through which the reduction of MTT occurs is not fully understood but most likely involves reaction with mitochondrial NADH and/or other intercellular reducing agents [69–71].

The MTT assay was conducted following the manufacturer's protocol, with minor modifications. Briefly, HUVECs were seeded at 2 × 10^4^ cells per well in 24-well plates and treated as required. 24 hours after a treatment, MTT (50 *μ*l; final volume of 0.25 mg/ml) was added to each well. The cells were incubated at 37°C for 4 h to ensure complete reduction of MTT to formazan crystals. The culture medium was carefully removed prior to addition of MTT solubilisation solution and gentle agitation for 10 min. Absorption (*λ* = 570 nm) was measured using a plate reader (Varioskan Flash, Thermo Fisher Scientific Ltd., Loughborough, UK). Background absorbance at *λ* = 630 nm was also measured for subsequent subtraction from *λ* = 570 nm readings. Experiments were repeated on 6–11 separate occasions, each in triplicate.

### 2.8. Superoxide Assay

Superoxide was measured using a fluorometric technique (ROS-ID Total ROS/Superoxide detection kit; Enzo Life Sciences Ltd.) that allows real-time measurement of (a) superoxide and (b) nonspecific ROS generation in cells. The kit contains a cell-permeable dye (orange probe) that reacts specifically with O_2_^∙−^ [72, 73], generating an orange fluorescent product that can be detected by a fluorescence plate reader equipped with standard orange filter sets (*λ* = 550/610 nm).

For this experiment, HUVECs were seeded at 2 × 10^4^ cells per well in 24-well plates. The cells were cotreated with pyrogallol (140 *μ*M) with or without delphinidin or gallic acid at concentrations of 1 nM, 1 *μ*M, and 100 *μ*M. Superoxide generation was measured at 4, 8, 12, and 24 h after treatment, following the manufacturer's protocol. Briefly, after the required amount of time, tissue culture medium was removed from the wells and discarded. HUVECs were washed immediately with 500 *μ*l/well of the supplied wash buffer. These steps reduce the likelihood of direct interference of the exogenous agents on the assay. Following wash buffer removal, 400 *μ*l/well of superoxide detection mix (2 *μ*l of superoxide detection reagent/10 ml of wash buffer) was added prior to incubation of plates in a humidified incubator (37°C, 5% CO_2_) and reading on a plate reader (*λ*_Ex_ = 550 nm and *λ*_Em_ = 610 nm) without removing the detection mix. The experiment was performed on 6–8 separate occasions, each in triplicate. Data from the four time points was integrated into an area under the curve for each experiment.

### 2.9. Total Glutathione Concentration, Catalase, and SOD Activity Assays

HUVECs were cultured in 6 well plates according to the method described above. The cells were cotreated with the superoxide radical generator, pyrogallol (180 *μ*M) and delphinidin or gallic acid, at concentrations that caused the most prominent protective effect against chemically induced oxidative stress (100 nM and 1 *μ*M).

GSH, catalase, and SOD assays were performed according to the manufacturer's protocol (Cayman Chemical Company). Total intracellular GSH concentration, SOD, and catalase activities were normalised to the amount of the protein present in cultured cells, determined using the Coomassie assay following the manufacturer's protocol (Thermo Fisher Scientific Ltd., Loughborough, UK). The experiment was performed on 6 separate occasions, each in duplicate.

### 2.10. Statistical Analysis

The results are expressed as mean ± SE. Statistical analysis (one-factor ANOVA with Dunnett's posttest, 2-factor ANOVA where applicable) was performed using GraphPad Prism software version 5.01 (GraphPad Software, San Diego, USA). *P* values of less than 0.05 were considered to be significant.

## 3. Results

### 3.1. Determination of Delphinidin Degradation Pattern in Tissue Culture Medium (37°C, pH 7.4) Spectrophotometry

**Figure 2:**
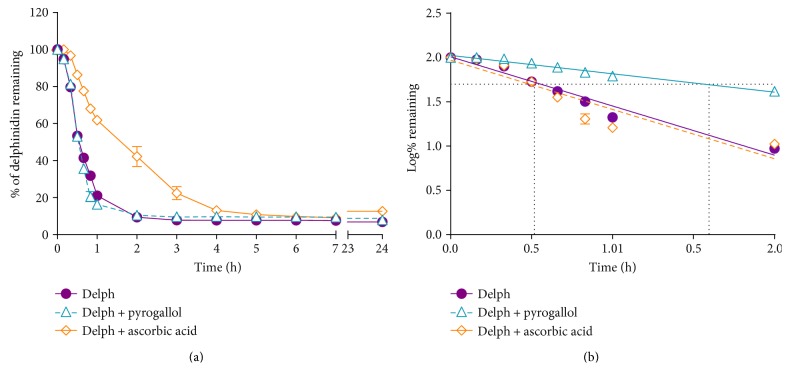
(a) Percent of delphinidin (Delph; 200 *μ*M at T_0_) remaining in tissue culture medium (pH 7.4, 37°C), when alone and in the presence of the oxidising agent (pyrogallol, 100 *μ*M) or the reducing agent/antioxidant, ascorbic acid (5 mM). (b) Semilogarithmic representation of percent of Delph remaining in solution; dotted lines indicate the respective half-lives in the presence and absence of ascorbic acid. The results are expressed as mean ± SEM (a) and mean only (b).

Delphinidin (200 *μ*M) was unstable in tissue culture medium (37°C, pH 7.4). The degradation process started immediately and was most rapid in the first hour (Figure 2(a)); a semilogarithmic plot indicated a linear relationship between log concentration and time, suggesting decay by first order kinetics (Figure 2(b)). Ascorbic acid (5 mM) slowed the degradation process, but the oxidant, pyrogallol, did not significantly affect the stability of delphinidin. The half-life was very short, (~30 min in the absence of ascorbic acid; Figure 2(a)); ~80% of delphinidin was lost in the first hour. Ascorbic acid increased the half-life to >1.5 h (Figure 2(a)).

### 3.2. Identification of Delphinidin Degradation Products in Tissue Culture Medium

**Figure 3:**
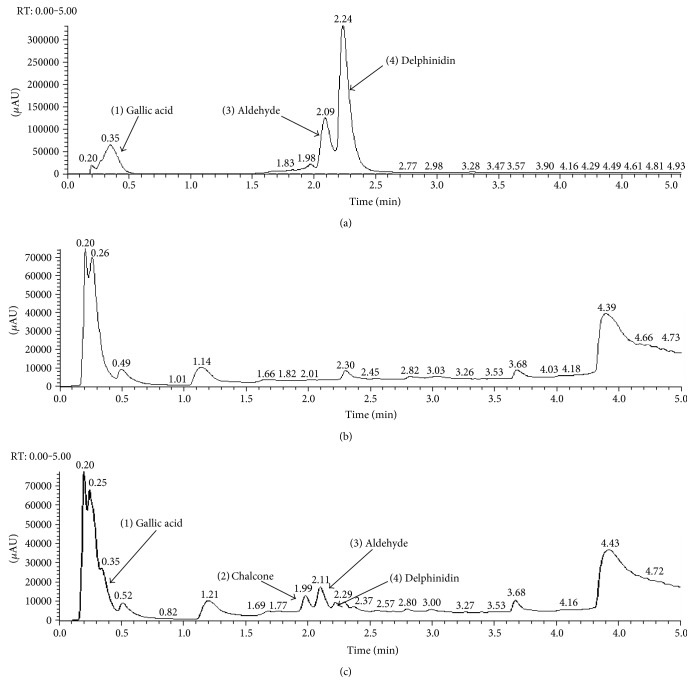
LC-MS chromatograms of sample containing (a) standards, (b) culture medium alone, and (c) delphinidin (100 *μ*M; pH 7.4, temperature 37°C) after 30 minute incubation.

Two delphinidin degradation products of relatively low molecular weight were identified in phenol red-free tissue culture medium (pH 7.4, temperature 37°C) after 30 min incubation, using LC-MS/MS: gallic acid (GA; peak 1) and phloroglucinol aldehyde (peak 3) (Figure 3). The parent compound (peak 4) was also still present at this time point. An unknown compound (peak 2) that was not present in the control samples was also detected. Based on the characteristics gathered from the LCMS/MS analysis (Table 1), the compound was putatively identified as chalcone on account of absorption maxima at *λ* ~ 220 and 320 nm [74]. Moreover, the peak contained a main ion at 321 (*m/z*) [M + H], which fragmented into two smaller ions at 303.03 and 152.97 (*m/z*), corresponding to delphinidin and aldehyde, respectively (Table 1).

### 3.3. Antioxidant Potential

The FRAP assay was only able to detect antioxidant activity in delphinidin and GA concentrations of ≥10 *μ*M (Figure 1(a)). There was a small (~5%) but significant higher antioxidant potential for delphinidin compared to gallic acid, as measured by FRAP (2-way ANOVA with Bonferroni posttest *P* < 0.001 for 10 and 100 *μ*M).

### 3.4. Measurement of Pro-/Antioxidant Properties (EPR Spectroscopy)

Both delphinidin and gallic acid exhibited oxygen-centred radical generating activity when present at 10 *μ*M and 100 *μ*M. Gallic acid also showed modest free radical scavenging properties at ≤1 *μ*M concentrations (Figure 1(c)). The radical-generating effect was significantly higher for delphinidin than gallic acid across the concentration range (Figure 1(c); 2-factor ANOVA, *P* < 0.05).

### 3.5. Effects of Delphinidin and Gallic Acid on Viability of HUVECs Determined by Trypan Blue Exclusion

**Figure 4:**
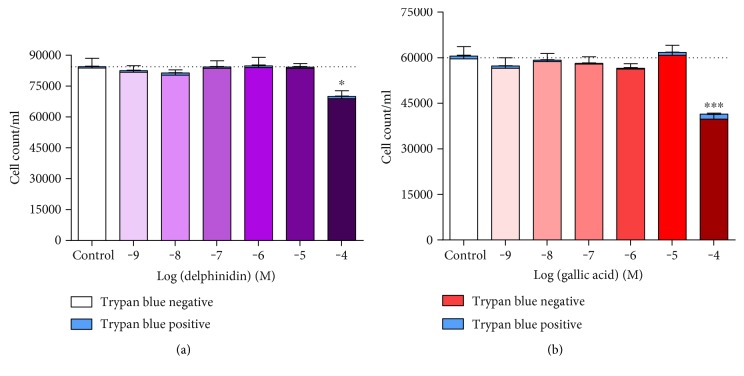
Number of Trypan blue-negative and Trypan blue-positive HUVECs after treatment (24 h) with increasing concentrations of (a) delphinidin (Del) and (b) gallic acid (GA), determined by Trypan blue exclusion. Values are shown as mean ± SEM (*n* = 5); ^∗^*P* < 0.05 and ^∗∗∗^*P* < 0.001 indicate the difference between Trypan blue-negative cells and the vehicle-treated control (one-way ANOVA with Dunnett's posttest).

Delphinidin and gallic acid did not have a significant impact on cell integrity at concentrations ≤ 10 *μ*M, as measured by Trypan blue exclusion assays. However, both agents at concentrations of 100 *μ*M caused a significant (~30 and 50%, resp.) reduction in Trypan blue-negative cells (Figure 4). Moreover, 100 *μ*M gallic acid caused a modest increase in Trypan blue-positive cells (~5%).

### 3.6. Effects of Delphinidin and GA on Viability of Pyrogallol-Induced Cell Death in HUVECs

**Figure 5:**
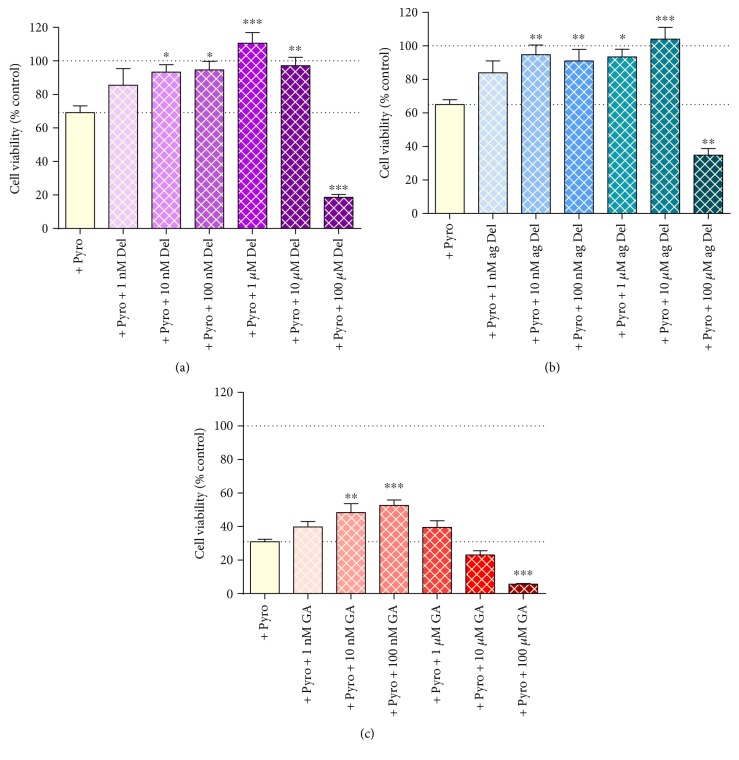
Cell viability, measured by MTT, in HUVECs cotreated with pyrogallol (140 *μ*M) and (a) delphinidin (Del), (b) aged delphinidin (ag Del), or (c) gallic acid (GA). Values are shown as mean ± SEM, (*n* = 7); ^∗^*P* < 0.05, ^∗∗^*P* < 0.01, and ^∗∗∗^*P* < 0.001, compared with the pyrogallol-treated cells (one-way ANOVA with Dunnett's posttest).

Pyrogallol induced a loss of cell viability (~30–70%; MTT assay) that was at least partially protected against by delphinidin, aged delphinidin, and GA at concentrations of ≤10 *μ*M (Figure 5). The effect was the clearest for delphinidin (Figure 5(a)) and aged delphinidin (Figure 5(b)), where cell viability was entirely protected by concentrations of 10 nM–10 *μ*M (peaking at ~1 *μ*M). 100 *μ*M delphinidin and aged delphinidin induced additional cytotoxic effects that were greater than those seen with pyrogallol alone; the additional effect was similar in amplitude to that seen with delphinidin in the absence of pyrogallol (Figure 4).

Gallic acid also significantly reduced pyrogallol-induced toxicity, but at even lower concentrations (10–100 nM; Figure 5(c)). The effect was less complete than for delphinidin and aged delphinidin, but it is important to note that the effect of pyrogallol alone was more pronounced in this particular set of experiments (~70% reduced viability compared to 30–40%). 100 *μ*M GA had a substantial additional cytotoxic effect, reducing cell viability to ~5% of control cells.

### 3.7. Effects of Delphinidin and Gallic Acid on Hydrogen Peroxide-Induced Cytotoxicity in HUVECs

**Figure 6:**
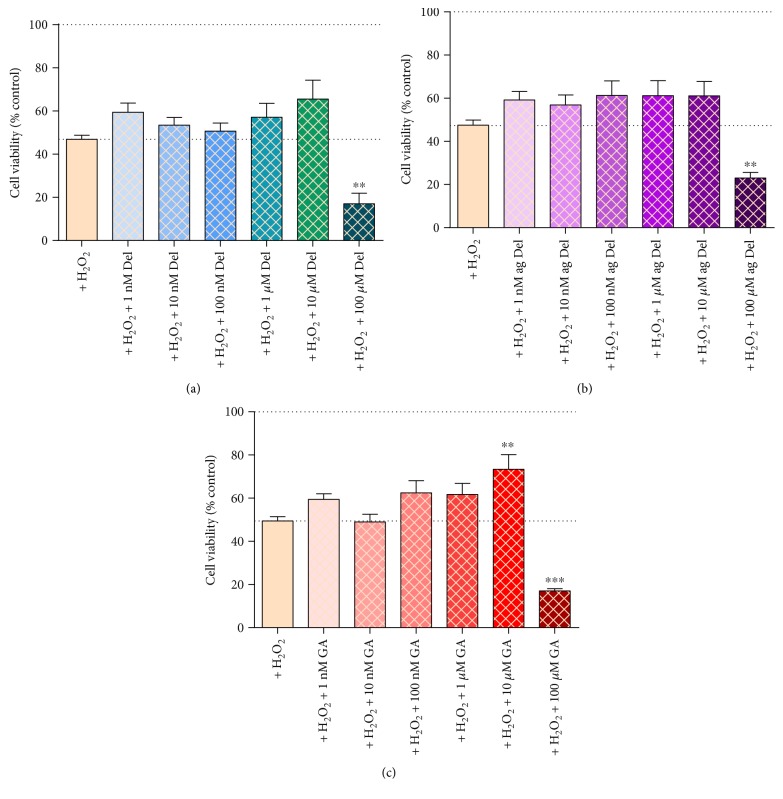
Cell viability, measured by MTT, in HUVECs cotreated with hydrogen peroxide (130 *μ*M) and (a) delphinidin- (Del-), (b) aged delphinidin- (ag Del-), or (c) gallic acid- (GA-) treated HUVECs. Values are shown as mean ± SEM (*n* = 6); ^∗∗^*P* < 0.01 and ^∗∗∗^*P* < 0.001 indicate the difference from oxidising agent treatment (one-way ANOVA with Dunnett's posttest).

Hydrogen peroxide induced loss of cell viability by ~55% (Figure 6). Only gallic acid (10 *μ*M) significantly protected against H_2_O_2_-induced attenuation (~40% protected; Figure 6(c)). There was a trend across all the phenolic treatments tested at 10 nM–10 *μ*M concentrations to induce a protective effect, but the findings were not statistically significant. In all cases, coincubation with phenolic treatments at 100 *μ*M with H_2_O_2_ exacerbated H_2_O_2_-induced cell death (~80% loss in viability; Figure 6).

### 3.8. Effects of Delphinidin and Its Degradation Products on the Viability of Pyocyanin-Induced Cytotoxicity in HUVECs

**Figure 7:**
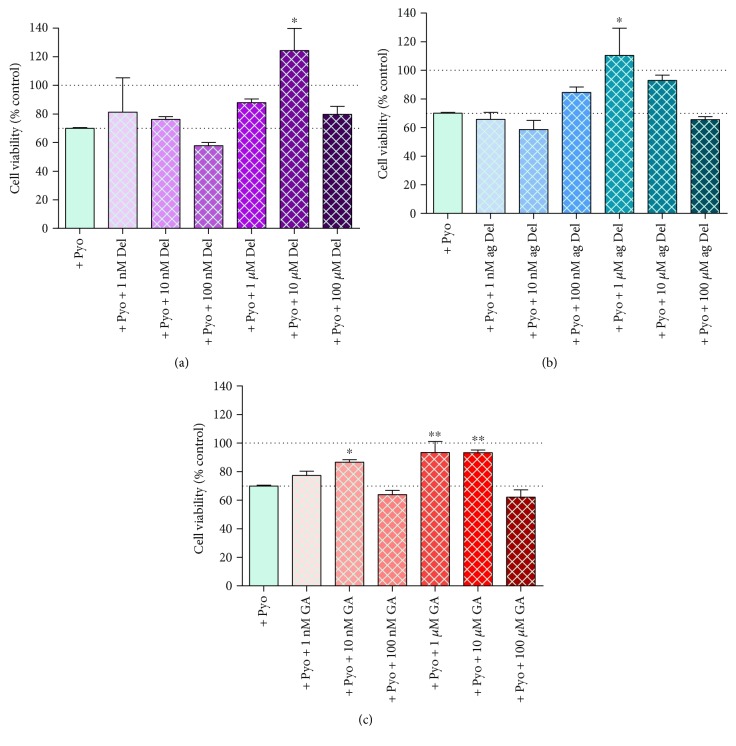
Cell viability, as measured by MTT assay, in HUVECs cotreated with pyocyanin (300 *μ*M) and (a) delphinidin (Del), (b) aged delphinidin (ag Del), and (c) gallic acid (GA). Values are shown as mean ± SEM (*n* = 4); ^∗^*P* < 0.05 and ^∗∗^*P* < 0.01 compared with the oxidising agent-treated cells (one-way ANOVA with Dunnett's posttest).

Pyocyanin reduced cell viability by ~30% (Figure 7). Delphinidin, aged delphinidin, and gallic acid offered protection to pyocyanin-treated cells, particularly at concentrations of 10 *μ*M and 1 *μ*M; the other tested concentrations failed to significantly protect cells against pyocyanin (Figure 7). Contrary to the data for H_2_O_2_ and pyrogallol-treated cells, there were no additional cytotoxic effects of 100 *μ*M concentrations of the phenolic treatments on pyocyanin-induced cell death.

### 3.9. Effect of Delphinidin and Gallic Acid on Endogenous Antioxidant Defences in Pyrogallol- (O_2_^∙−^) Treated HUVECs

#### 3.9.1. Effect of Pyrogallol and Phenolics on Intracellular Superoxide

**Figure 8:**
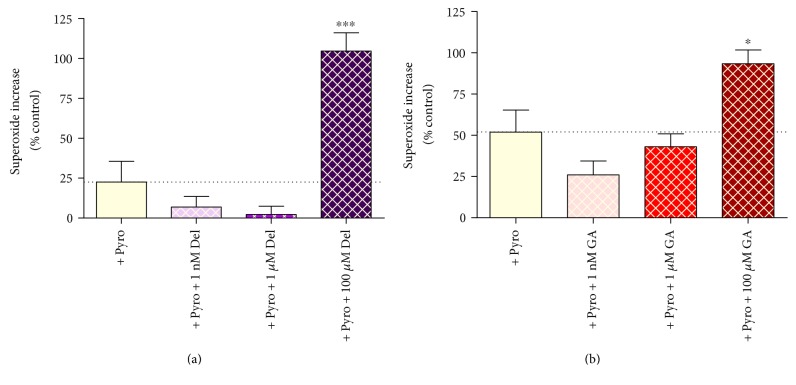
Effect of pyrogallol alone and (a) pyrogallol + delphinidin (Del) or (b) pyrogallol + gallic acid (GA) on intracellular superoxide in HUVECs. Values are shown as mean ± SEM (*n* = 6–8). ^∗^*P* < 0.05 and ^∗∗∗^*P* < 0.001 compared to pyrogallol alone (one-way ANOVA with Dunnett's posttest).

Treatment of HUVECs with exogenous pyrogallol induced an increase in intracellular superoxide, measured using an intracellular fluorescent probe (Figures 8(a) and 8(b); +86%); total ROS was not significantly affected (results not shown). Cotreatment of cells with a moderate (1 *μ*M) concentration of either delphinidin (Figure 8(a)) or gallic acid (Figure 8(b)) trended towards a reduction in intracellular superoxide concentration, while cotreatment with 100 *μ*M delphinidin or gallic acid induced substantial increases in intracellular superoxide (a further increase of +73% or +41%, resp., beyond the pyrogallol-induced increase).

#### 3.9.2. Intracellular Total GSH

**Figure 9:**
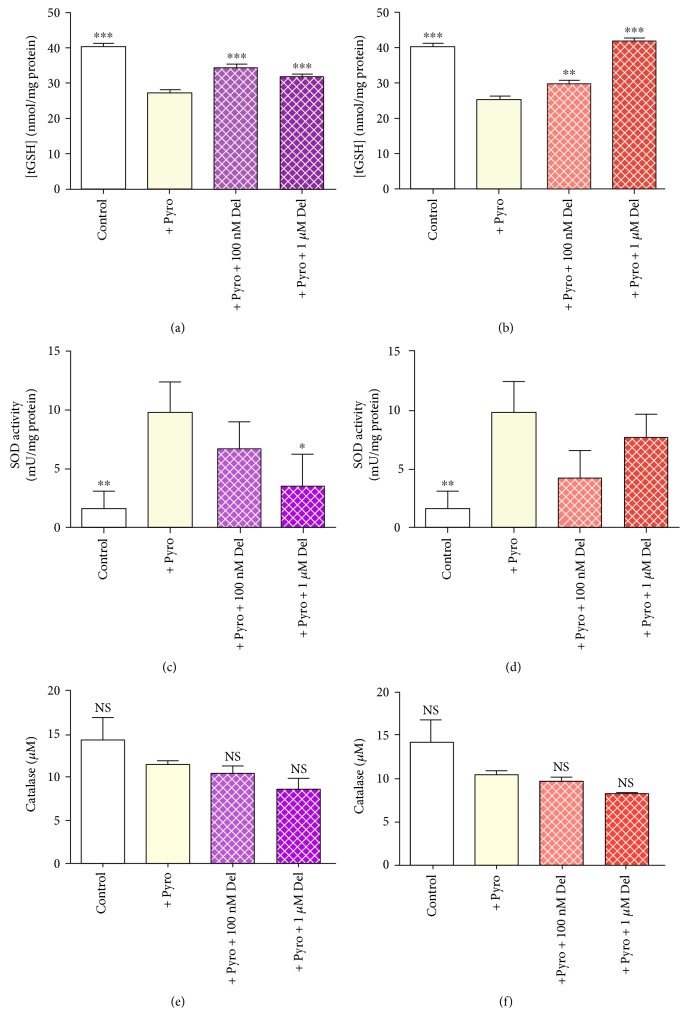
Total GSH concentration (a, b), SOD activity (c, d), and catalase activity (e, f) in HUVECs treated with delphinidin (Del; a, c, and e) and gallic acid (GA; b, d, and f) at concentrations of 1 *μ*M and 100 nM, measured at 24 h. Values are shown as mean ± SEM (*n* = 6); ^∗^*P* < 0.05, ^∗∗^*P* < 0.01, and ^∗∗∗^*P* < 0.001 indicate the difference from oxidising agent treatment (one-way ANOVA with Dunnett's posttest).

Pyrogallol (180 *μ*M) was associated with a depression of total cellular GSH in HUVECs by ~35%, after 24 h (*P* < 0.001, compared to control; Figures 9(a) and 9(b)). Delphinidin (100 nM and 1 *μ*M) significantly protected against pyrogallol-associated depression of total GSH in HUVECs at 24 h (*P* < 0.001, for both; Figure 9(a)), although the concentration of GSH did not reach the same level as that of cells not treated with pyrogallol (control). Gallic acid at 100 nM had an effect similar in magnitude to that of delphinidin, while gallic acid at 1 *μ*M concentration fully protected against oxidant-induced depression of total glutathione (Figure 9(b)).

#### 3.9.3. SOD Activity

Superoxide dismutase (SOD) activity in HUVECs was low (<5 mU/mg protein) in untreated cells. Pyrogallol notably enhanced SOD activity (~10 times compared to control; Figures 9(c) and 9(d)). Neither delphinidin (Figure 9(c)) nor GA (Figure 9(d)) enhanced SOD activity beyond that seen with pyrogallol alone; indeed, the trend shown with both agents was to actively depress pyrogallol-induced SOD activity in this model. The specificity of the assay for SOD activity was confirmed by testing equivalent HUVEC extracts treated with pyrogallol with KCN immediately prior to the assay. KCN inhibited SOD activity by ~87%, indicating a strong specificity for SOD (*n* = 4; data not shown).

#### 3.9.4. Catalase Activity

Pyrogallol treatment (180 *μ*M) did not significantly change catalase activity in HUVECs at 24 h after treatment. Likewise, co-treatment with this oxidising agent and either phenolic compound failed to have any significant impact on catalase activity in the cells (Figures 9(e) and 9(f)).

## 4. Discussion

This study found that the anthocyanin, delphinidin, rapidly degrades under physiological conditions and fails to offer substantial antioxidant activity *in vitro* at concentrations relevant to oral bioavailability. Nevertheless, it effectively protects cultured endothelial cells against chemically induced oxidative stress. The protective effects of delphinidin were hormesic in profile, peaking at ~1 *μ*M. The key degradation product from delphinidin, gallic acid (GA), shared many of the antioxidant protective characteristics of the parent compound. Both delphinidin and gallic acid induced an increase in total intracellular GSH but did not increase activity of either SOD or catalase. The inference of these findings is that the antioxidant protective effects of delphinidin might not be mediated by direct antioxidant activity and do not necessarily require the presence of the parent compound. They are, however, associated with increased intracellular GSH that is as likely to be triggered by GA as by delphinidin itself. This association does not unequivocally identify GSH as the cause of the effect, but it is one explanation.

### 4.1. Stability of Delphinidin

Delphinidin is more stable under physiological conditions when present as a glycoside [75]. However, its stability declines rapidly when deprived of the sugar moiety during transit through the gut. Our results indicate that delphinidin aglycone is not stable under physiological conditions, with a short half-life of ~30 min. Ascorbic acid offers some protection against decomposition, but the half-life is still <2 h in the presence of this antioxidant.

LC-MS/MS data indicated that delphinidin degraded spontaneously to gallic acid and phloroglucinol aldehyde under physiologically relevant conditions in tissue culture medium. The fact that both degradation products appeared in the solution after only 30 minutes indicates the rapidity of the process and confirms previous observations [39, 41]. The presence of the intermediate degradation product, chalcone, is an indicative that the chemical process involved is similar to that previously described [39, 41, 56]. From a bioavailability perspective, these data imply that delphinidin, stripped of its sugar in the gut, is unlikely to persist for long in the blood or in tissue. It is likely that the more stable products of delphinidin decomposition and metabolism, including gallic acid, are more likely to have the opportunity to mediate any protective effects that are typically attributed to delphinidin.

### 4.2. *In Vitro* Anti- and Prooxidant Effects of Delphinidin and Gallic Acid

FRAP analysis is only detected reducing activity for delphinidin and gallic acid at concentrations of ≥10 *μ*M; although the effect was significantly greater for delphinidin than gallic acid, the absolute difference was only ~5%. In interpreting these data, it is important to highlight that FRAP, along with many of the other *in vitro* “antioxidant” assays, actually measures reducing power, rather than free radical scavenging. In addition, this technique will not identify any prooxidant effects. EPR spectrometry exclusively detects free radicals [59] and, through use of a selective spin trap for oxygen-centred radical generation, can provide a direct measurement of radical-mediated oxidising potential. The EPR data generated showed that both delphinidin and gallic acid exhibit prooxidant activities at ≥10 *μ*M, with some modest scavenging effects of oxygen-centred radical species generated spontaneously in tissue culture medium at lower concentrations. The prooxidant finding is not without precedent: phenolic compounds, including gallic acid, have been previously shown to have prooxidant properties [43, 44], possibly via reduction of transition metal ions and consequent induction of Fenton chemistry [59]. Several research groups [44, 75, 76] have reported that phenolic compounds, and gallic acid in particular, can oxidise readily in tissue culture medium and produce free radicals, such as superoxide radical (O_2_^∙−^), H_2_O_2_, and quinones. Indeed, autoxidation of phenolic compounds, and consequential free radical generation, could explain their cytotoxicity towards cancer cells at high concentrations [44]. What is clear from the FRAP and EPR data is the complexity that exists when dealing with phenolic compounds in relation to their reducing capacity, pro- and antioxidant activity. Certainly, the concept that concentration-dependent reducing power (FRAP) translates into antioxidant capacity *in vivo* does not hold on account of a concurrent, counterintuitive increase in oxygen-centred free radical generation; if anything, the EPR data indicated that only gallic acid at a concentration of ~1 *μ*M shows signs of a modest direct antioxidant effect.

### 4.3. Cytotoxicity and Protective Effects of Delphinidin and Gallic Acid in HUVECs

Physiologically relevant concentrations (≤10 *μ*M) of delphinidin and gallic acid did not induce cell death in HUVECs in the absence of experimental oxidative stress. However, at supraphysiological concentrations (100 *μ*M), capable of powerful reducing effects and concomitant oxygen-centred free radical production, there was a substantial cytotoxic effect measured by a loss of cell membrane integrity (primary or secondary necrosis). That the threshold for cytotoxicity coincides with detectable reducing power and free radical generation might suggest that cytotoxicity is driven by the oxidant-free radical production, in turn mediated by reduced metal ion-mediated Fenton chemistry. Whatever the mechanism, however, it is clear that these phenolic agents become toxic at concentrations above ~10 *μ*M, which could explain why they are largely excluded in the gut and why plasma concentrations are maintained below ~10 *μ*M.

### 4.4. Phenolic Compounds Protect against Oxidative Stress-Induced Cell Death: A Hormesic Relationship

Delphinidin, aged delphinidin, and GA had a substantial protective effect against chemically induced oxidative stress in HUVECs. The degree of protection was specific to the oxidant used; it was most pronounced in HUVECs exposed to superoxide radical generated from pyrogallol and via oxidative stress on account of pyocyanin-induced cell death. Protection was concentration dependent: for pyrogallol-treated cells, 100 nM and 1 *μ*M concentrations had the most pronounced beneficial effects, but very low concentrations (10 nM) also offered significant protection. In the case of both delphinidin and aged delphinidin, protection was sufficient to fully restore oxidant-induced loss of cell viability. The effectiveness of GA was significantly weaker compared to that of delphinidin and aged delphinidin, but this might be due to the fact that the extent of cell death induced by pyrogallol alone in the GA experiment was considerably higher than that in the delphinidin and aged-delphinidin experiments. In pyocyanin-treated cells, optimal effects were seen at 1–10 *μ*M, and for H_2_O_2_, only 10 *μ*M GA showed partial protective effect. While the pattern of effects was similar for all of the test agents, there were subtle differences in the optimal concentrations required for each and the relative effectiveness against the different oxidant stressors. In general, the optimal concentration for GA was ~10-fold lower than that for delphinidin and aged delphinidin, suggesting that treatment throughout 24 h with this metabolic product is more efficacious than initial treatment with delphinidin, despite the likely degradation of the latter to ultimately generate gallic acid. The data strongly suggest that delphinidin or indeed the unstable intermediate chalcones are not a requirement for antioxidant protection in this model.

Data involving direct measurement of intracellular superoxide using an intracellular fluorescent probe supported the concept that both delphinidin and GA at 100 *μ*M actively induce intracellular oxidative stress, suggesting that the cytotoxic effects seen in the cells are associated with oxidative stress. Meanwhile, the trend for low (1 nM) and intermediate concentrations was to quench intracellular superoxide, in keeping with the hormesic relationships seen through measurement of cell death in this model.

The concentration range chosen for this study embraces those relevant to *in vivo* bioavailability (100 nM–~1 *μ*M) [12, 14, 33, 37]. However, in order to obtain a better insight into the effectiveness of phenolic compounds, higher concentrations were included in the experimental design. Though the protective effects of the test agents were concentration dependent, the relationship did not follow the traditional sigmoidal log-concentration response relationship in which increasing effect (antioxidant protection in this case) increases with concentration and then reaches plateau. Instead, it was found that, whereas low to intermediate concentrations showed protective effects, the benefit was not seen with 100 *μ*M delphinidin, aged delphinidin, or gallic acid. Indeed, in the cells treated with pyrogallol or H_2_O_2,_ the cytotoxic effect of these agents is clearly additive to that caused by the chemical oxidant. The phenomenon where the same agent can be harmful in higher concentrations and beneficial at lower concentrations is called hormesis [77] and has previously been described for resveratrol, where a protective effect at low doses but an adverse effect at higher doses is observed [78]. Delphinidin and its degradation products display classic hormesis in HUVECs in the presence of an oxidant (particularly superoxide induced by pyrogallol or from pyocyanin-induced oxidative stress and cell death). Optimal benefit coincided with the concentrations that are likely to be available from dietary ingestion.

The results not only suggest that the positive attributes of polyphenolic compounds are overrated but also act as a reminder that *natural* does not automatically mean *safe* and *more* does not necessarily mean *better* [11]. It should be emphasised, however, that the log relationship of concentration with effect, coupled with the highly effective screening of phenolic compounds in the gut, ensures that levels of exposure that might generate a toxic effect are unachievable through diet. It would become an important consideration, however, that polyphenolic compounds should be considered for intravenous delivery or incorporation into implantable devices.

### 4.5. Impact of Delphinidin and Gallic Acid on Intracellular Defences—Secondary Antioxidant Effects

Total intracellular GSH concentrations increased in pyrogallol-treated HUVECs in response to both delphinidin and gallic acid present at 100 nM–1 *μ*M; the effect of gallic acid was sufficiently powerful to totally prevent pyrogallol-induced depression of total GSH. It is important to recognise that the observed association between protection by GA and protection of GSH cannot in itself establish that an increase in intracellular GSH is responsible for the protective effects of gallic acid—it could equally infer that gallic acid or delphinidin offers direct antioxidant protection resulting in protection of GSH. However, we consider the latter explanation to be unlikely on account of the lack of antioxidant activity of gallic acid at the relevant concentration (1 *μ*M) in any of the antioxidant assays used. In addition, the effect seen was on *total* GSH concentration not the balance of reduced/oxidised GSH composition, inferring modulation of GSH synthesis/breakdown rather than antioxidant protection. Should phenolic induction of GSH be confirmed, the concept is intriguing because it could drive a substantial amplification of antioxidant capacity: the impact of 1 *μ*M gallic acid on total cellular antioxidant capacity would be negligible, but through this process, the increase in intracellular GSH could be in the millimolar range.

This result contradicts those of previous studies in which both delphinidin [78] and gallic acid [79, 80] contributed significantly to GSH depletion and an increase in ROS production. However, the concentrations used in the previous studies were substantially higher (25–100 *μ*M and 10–400 *μ*M for delphinidin and gallic acid, resp.)—levels at which our data suggest that both delphinidin and gallic acid are capable of generating oxygen-centred free radicals. These discrepancies highlight the importance of close attention to the concentration of antioxidants applied in cell culture and their relevance to *in vivo* bioavailability.

Similar changes were not found for the key antioxidant defence enzymes that we investigated (SOD and catalase), suggesting a fairly targeted impact of these treatments. This conclusion is in line with a recent study [81], in which gallic acid was found to cause an increase in GST-alpha 3 without having a significant impact on SOD activity. Likewise, gallic acid increased activity of rat liver microsomal glutathione S-transferase MGST-1, but only in the absence of catalase and SOD [82].

The underlying mechanism by which delphinidin and gallic acid increase intracellular total GSH is not yet known, but upregulation of the rate-limiting enzyme responsible for GSH synthesis, glutamyl cysteine ligase (GCL), is a credible option. Polyphenols are known to activate the heterodimers of NF-E2-related factors 2 (Nrf2)/antioxidant-responsive element (ARE) pathway, leading to induction of detoxifying enzymes [49, 83], including GCL amongst others [84], and we postulate that gallic acid might activate a similar pathway.

### 4.6. Mechanism and Implications

There is a growing body of evidence that phenolic compounds are treated as xenobiotics by cells [34, 85]. Therefore, the extensive metabolism that they are exposed to, coupled with their low bioavailability, may be a result of the natural defence against a potentially toxic insult. This concept would explain the relatively low concentrations of (poly)phenolics found in plasma (100 nM–10 *μ*M), which is not dramatically affected by ingestion of phenolic-rich food [2, 14, 54]. The small amount of parent polyphenols that overcome the defence mechanism, together with the simple phenolic compounds that represent degradation products, imposes a mild noxious effect, driving Nrf-2/ARE-1-mediated upregulation of intracellular defence enzymes and xenobiotic-metabolising enzymes, leading to the observed antioxidant and cytoprotective effect.

The results of this study provide support for the view that delphindin—and potentially other berry-derived polyphenols—has the capability to protect endothelial cells against oxidative stress, perhaps via an indirect route. In contradiction to common opinion, the protection offered does not appear to be related to direct antioxidant properties of the polyphenol itself; instead, modulation of intracellular defence mechanisms is a possible alternative mode of action. Moreover, this study suggests that degradation products are likely to be responsible for the observed biological activities, rather than the parent compound itself. This has profound implications for *in vivo* bioavailability studies, suggesting that our attention might have been previously misplaced and that we should instead measure phenolic metabolites, which can also confer bioactivity and might be present at concentrations higher than the parent compound(s).

Dietary polyphenols have health-promoting qualities, but their mode of action still remains equivocal. A full understanding of the bioactive agents *in vivo* and their mode of action will help to shape public health advice. There are also profound consequences of our findings for research practice, particularly with respect to the relevance of *in vitro* antioxidant (reducing capacity) testing as an indication of putative health benefits of food extracts or polyphenolic compounds.

## 5. Conclusions

This research adds to the growing literature that explains the apparent disparity between the very low bioavailability and rapid metabolism of complex polyphenols and their ability, nevertheless, to evoke a powerful antioxidant effect. At high concentrations, delphinidin and its major metabolite, GA, have the potential to be toxic, but at subtoxic concentrations, they can protect against oxidative stress through a mechanism that is associated with increased glutathione. The implications are that antioxidant activity of polyphenols might not have any bearing on their ability to protect cells against oxidative stress; instead, it is the capability of the bioavailable metabolites to stimulate antioxidant defence pathways that likely drives the protective effect, in a manner unrelated to the *in vitro* reducing capacity of the parent molecule [55].

## Figures and Tables

**Table 1:** Chromatographic and mass spectrometric properties of delphinidin and its degradation products.

Peak number	RT (min)	Abs max (nm)	[M + H]	[M − H]	Putative identification
1	0.43	220, 270	N/A	169.01	Gallic acid
2	2.02	224, 320	321.06	319.04	Chalcone
3	2.13	227, 291	155.03	153.02	Aldehyde
4	2.30	280, 529	303.05	N/A	Delphinidin
